# Clinical characteristics and acute complication of COVID-19 patients with diabetes: a multicenter, retrospective study in Southern China

**DOI:** 10.3389/fendo.2023.1237832

**Published:** 2023-08-14

**Authors:** Xiao-ying Zhou, Shao-feng Huang, Jun-xu Lin, Hai-ni Zhi, Lu Xiao, Xiang-zhu Wang, Kai-heng Guo, Lin Zhou, Tao Long, Hui-min You, Ming-run Lin, Xiang-ya Luo, Wei-ping Sun, Chun-ping Zeng

**Affiliations:** ^1^Department of Endocrinology and Metabolism, The Fifth Affiliated Hospital of Guangzhou Medical University, Guangzhou, China; ^2^Department of Endocrinology, The Affiliated Loudi Hospital, Hengyang Medical School, University of South China, Hengyang, Hunan, China; ^3^Department of Endocrinology and Metabolism, Loudi Central Hospital, Loudi, China

**Keywords:** COVID-19, diabetes, clinical characteristics, acute complications, renal insufficiency

## Abstract

**Aims:**

This study aims to describe the clinical characteristics, laboratory data and complications of hospitalized COVID-19 patients with type 2 diabetes mellitus (T2DM) since epidemic prevention and control optimization was adjusted in December 2022 in China.

**Methods:**

This retrospective multicenter study included 298 patients with confirmed type 2 diabetes mellitus with or without COVID-19. We collected data from the first wave of the pandemic in The Fifth Affiliated Hospital of Guangzhou Medical University, Loudi Central Hospital and The First People’s Hospital of Xiangtan from December 1, 2022 to February 1, 2023. We extracted baseline data, clinical symptoms, acute complications, laboratory findings, treatment and outcome data of each patient from electronic medical records.

**Results:**

For among 298 hospitalized patients with type 2 diabetes, 136 (45.6%) were COVID-19 uninfected, and 162 (54.4%) were COVID-19 infected. We found that the incidence of cough, fatigue, fever, muscle soreness, sore throat, shortness of breath, hyposmia, hypogeusia and polyphagia (all p<0.01) were significantly higher in the exposure group. They showed higher levels of ketone (p=0.04), creatinine (p<0.01), blood potassium (p=0.01) and more diabetic ketoacidosis (p<0.01). Patients with COVID-19 less use of metformin (p<0.01), thiazolidinediones (p<0.01) and SGLT2 (p<0.01) compared with patients without COVID-19.

**Conclusion:**

COVID-19 patients with diabetes showed more severe respiratory and constitutional symptoms and an increased proportion of hyposmia and hypogeusia. Moreover, COVID-19 patients with diabetes have a higher incidence of acute complications, are more prone to worsening renal function, and are more cautious about the use of antidiabetic drugs.

## 1 Introduction

Since the optimization and adjustment of epidemic prevention and the orderly restoration of production and living in December 2022, COVID-19 has started the first wave of the pandemic in China. At present, SARS-CoV-2 Omicron BA.5.2 variants is progressively displacing other variants in southern CHINA, which displays a higher transmissibility than other Omicron subvariants (1). Since China no longer requires nucleic acid detection of SARS-CoV-2, the infection rate of the novel coronavirus is not available. The Centers for Disease Control and Prevention (CDC) in Sichuan (a province in southern CHINA) estimated the prevalence of COVID-19 approximately 65.5% as of December 26, 2022. According to the big data model of the National School of Development, as of January 11, 2023, the cumulative infection rate of COVID-19 in China is approximately 64%, and the cumulative number of infected people is approximately 900 million.

Several studies have reported that COVID-19 patients are more susceptible to type 2 diabetes, and the severity and mortality of COVID-19 in diabetes patients are higher than those in patients without diabetes (2, 3). In China, the reported prevalence of diabetes in patients with COVID-19 is similar to the national prevalence of T2DM, approximately 11% (4, 5). In New Delhi, India, the prevalence of diabetes among people with COVID-19 is 47%, which is far higher than the prevalence of T2DM in this region (6). Although the prevalence of diabetes among COVID-19 patients varies by region, studies have reported that the proportion of COVID-19 patients with diabetes is relatively high (7, 8). Moreover, COVID-19 patients with diabetes might be at increased risk of acute metabolic complications, especially an increase in diabetic ketoacidosis during the COVID-19 pandemic (9). The patients with diabetes also had abnormal blood glucose levels and increased the dose of insulin during hospitalization, which indicated their poor glycemic control (10). The increased prevalence, severity, and complications of type 2 diabetes in COVID-19 patients may be related to human angiotensin-converting enzyme 2 (ACE2) and transmembrane serine protease 2 receptors, which are expressed on pancreatic beta cells (11). SARS-CoV-2 infection has been shown to reduce insulin secretion levels and induce pancreatic β cell apoptosis (12).

We conducted a multicenter retrospective study in southern China, aiming to compare the basic information, laboratory examinations, clinical symptoms, acute complications, and medication in patients with diabetes with or without COVID-19, and ascertain the impact of COVID-19 on diabetes.

## 2 Materials and methods

This retrospective multicenter study included 298 patients with confirmed type 2 diabetes mellitus with or without COVID-19. We collected data from the first wave of the pandemic in The Fifth Affiliated Hospital of Guangzhou Medical University, Loudi Central Hospital and The First People’s Hospital of Xiangtan from December 1, 2022 to February 1, 2023. This research was conducted with approval from the Fifth Affiliated of Guangzhou Medical University Research Ethics Committee. (GYWY-L2023-64).

Patients fulfilling the following criteria were included in this study: physician diagnosis of T2DM and aged over 18 years. Gravidas and patients with severe multi-organ dysfunction were excluded. We divided all patients into two groups depending on whether they were confirmed with COVID-19 after novel coronavirus nucleic acid testing or novel coronavirus antigen detection. All the enrolled patients were hospitalized due to poor blood glucose control or new-onset diabetes, among which the T2DM patients with COVID-19 had been clearly infected before hospitalization.

We extracted baseline data, clinical symptoms, acute complications, laboratory findings, treatment and outcome data of each patient from electronic medical records. All data collected were reviewed by the research team and double checked by experienced physicians. Patients with missing data or unknown medical records were excluded.

We used SPSS (version 26.0; IBM) statistical software to analyze and process the data. Quantitative data were expressed as x±s and differences between groups were compared using independent sample t-test if they were normally distributed. Quantitative data that did not conform to a normal distribution and ordinal data were expressed as medians or quartile ranges and differences between groups were compared using nonparametric tests. Qualitative data were described by frequency or percentage and differences between groups were compared using the χ^2^ test. For all the statistical analyses, a p-value < 0.05 was considered statistically significant.

## 3 Results

A total of 298 hospitalized patients with type 2 diabetes were included in this retrospective study. Among these patients, 136 (45.6%) were COVID-19 uninfected and 162 (54.4%) were COVID-19 infected, respectively. Of all hospitalized patients, the median age was 64 years (IQR, 49-67) and the median duration of diabetes was 8.0 (IQR, 3.0-14.0). Compared with patients without COVID-19, patients with COVID-19 infected had older age (68.5 [IQR, 59.0-76.0]), longer diabetes duration (10.0 [IQR, 4.0-19.0]), and had no significant differences in either systolic blood pressure or diastolic blood pressure. The most common symptoms were cough (143 [48.0%]), fatigue (132 [44.3%]), fever (61[20.5%]), dizziness (77 [25.8%]), shortness of breath (61 [20.5%]), nausea (67 [22.5%]), polyuria (75[25.2%]) and polydipsia (132 [44.3]) at illness onset. Less common symptoms included muscle soreness, runny nose, sore throat, chest distress, diarrhea, hyposmia, hypogeusia, polyphagia and weight loss. We found that the incidence of cough (37[27.2%] vs 106[65.4%]; p<0.01), fatigue (37[27.3%] vs 95[58.6%]; p<0.01), fever (16[11.8%] vs 45[27.8%]; p<0.01), muscle soreness (3[2.2%] vs 22[13.6%]; p<0.01), sore throat (8[5.9%] vs 23[14.2%]; p=0.02), shortness of breath (18[13.2%] vs 43[26.5%]; p<0.01), hyposmia (0[0%] vs 14[8.6%]; p<0.01), hypogeusia (1[0.7%] vs 17[10.5%]; p<0.01) and polyphagia (1[0.7% vs 25[15.4%]; p<0.01) were significantly higher in the exposure group than in the non- exposure group (**Table 1**).

**Table 1:** Baseline characteristic and clinical signs and symptoms of patients infected with COVID-19.

			No.(%)		
		Total (n=298)	COVID-19 uninfected (n=136)	COVID-19 infected (n=162)	p value
Age, Median (IQR), years		64.0 (54.0-74.0)	60.0 (51.2-70.0)	68.5 (59.0-76.0)	0.000058
Diabetes duration, Median (IQR), years		8.0 (3.0-14.0)	7.0 (2-10)	10.0 (4.0-19.0)	0.003054
Systolic blood pressure, Median (IQR), mm Hg		133.0 (122.0-149.0)	133.0 (123.3-149.8)	133.5 (120.8-148.3)	0.556
Diastolic blood pressure, Median (IQR), mm Hg		78.0 (70.0-87.0)	77.5 (70.0-88.0)	79.0 (70.0-86.3)	0.724
Signs and symptoms	Cough	143 (48.0)	37 (27.2)	106 (65.4)	5.0998E-11
	Fatigue	132 (44.3)	37 (27.3)	95 (58.6)	5.5597E-8
	Fever	61 (20.5)	16 (11.8)	45 (27.8)	2.2877E-10
	Dizziness	77 (25.8)	44 (32.4)	33 (20.4)	0.696413
	Muscle soreness	25 (8.4)	3 (2.2)	22 (13.6)	0.000014
	Runny nose	4 (1.3)	1 (0.7)	3 (1.9)	0.150758
	Sore throat	31 (10.4)	8 (5.9)	23 (14.2)	0.019388
	Chest distress	53 (17.8)	22 (16.2)	31 (19.1)	0.506485
	Shortness of breath	61 (20.5)	18 (13.2)	43 (26.5)	0.004637
	Nausea	67 (22.5)	27 (19.9)	40 (24.7)	0.319801
	Diarrhea	25 (8.4)	6 (4.4)	19 (11.7)	0.023482
	Hyposmia	14 (4.7)	0 (0)	14 (8.6)	0.000455
	Hypogeusia	18 (6.0)	1 (0.7)	17 (10.5)	0.000438
	Polydipsia	132 (44.3)	58 (42.6)	74 (45.7)	0.600316
	Polyphagia	26 (8.7)	1 (0.7)	25 (15.4)	0.000297
	Polyuria	75 (25.2)	31 (22.8)	44 (40.3)	0.387774
	Weight loss	43 (14.4)	17 (12.5)	26 (16.0)	0.385904

The laboratory test results and the incidence of acute complications at admission are shown in **Table 2**. In all the patients, glycosylated hemoglobin and fasting plasma glucose were above the normal range, while the values of other laboratory indicators were within the normal range. Compared to non-exposure group, the exposure group showed higher levels of ketone (0.2 [IQR, 0.1-0.3] vs 0.2 [IQR, 0.1-0.9]; p=0.04), creatinine (69.3 [IQR, 53.7-89.3] vs 86.5 [IQR, 63.3-121.0]); p<0.01), blood potassium (4.0[IQR, 3.7-4.3] vs 4.2[IQR, 3.7-4.6], p=0.01) and more diabetic ketoacidosis (8[5.9%] vs 34[21.0]; p<0.01). These laboratory data indicated that the COVID-19 patients with diabetes are more likely to develop diabetic ketoacidosis and are at greater risk of developing renal insufficiency than those without COVID-19 infection (**Table 2**).

**Table 2:** Comparison of laboratory parameters and complication between COVID-19 infected and uninfected diabetic patients.

		Median (IQR)		
	Normal range	COVID-19 uninfected (n=136)	COVID-19 infected (n=162)	p value
HbA1c, %	4.0-6.0	9.1 (7.5-11.2)	8.3 (7.4-11.0)	0.440
FPG, mmol/L	3.9-6.1	9.2 (6.7-11.2)	8.4 (6.1-12.5)	0.284
Ketone, mmol/L	0-0.3	0.2 (0.1-0.3)	0.2 (0.1-0.9)	0.035
WBC, ×10^9^/L	3.5-9.5	6.9 (5.4-8.8)	6.4 (4.7-9.9)	0.274
Neut, ×10^9^/L	1.8-6.3	4.11 (3.17-6.15)	4.6 (3.0-7.0)	0.492
Hemoglobin, g/L	115-150	131.0 (112.3-142.0)	125.0 (111.8-140.0)	0.297
Creatinine, umol/L	53-106	69.3 (53.7-89.3)	86.5 (63.3-121.0)	0.001
ALT, U/L	≤40	20.4 (13.4-31.6)	21.0 (14.1-31.3)	0.891
AST, U/L	≤41	25.4 (17.7-33.2)	29.6 (20.5-41.0)	0.559
Blood potassium, mmol/L	3.5-5.5	4.0 (3.7-4.3)	4.2 (3.7-4.6)	0.013
Blood sodium, mmol/L	135-145	138.2 (136.0-141.0)	137.4 (134.3-141.6)	0.455
Blood chlorine, mmol/L	96-168	103.1 (100.3-105.3)	101.8 (98.9-105.8)	0.576
Diabetic ketoacidosis, No. (%)	–	8 (5.9)	34 (21.0)	0.000194
Diabetic hyperosmolar coma, No. (%)	–	1 (0.7)	1 (0.6)	0.150758

HbA1c, Glycosylated Hemoglobin; FPG, Fasting Plasma Glucose; WBC, White cell count; Neut, Neutrophil count; ALT, Alanine Aminotransferase; AST, Aspartate Aminotransferase.

During hospitalization, most patients were treated with insulin (192[64.1%]), followed by metformin (98[32.9%]), SGLT2 (71[23.8%]), glucosidase inhibitors (48[15.4%]), DPP4 (41[13.8%]), thiazolidinediones (30[10.1%]), GLP1 (20[6.7%]), sulfnoylureas (7[2.3%]) and glinides (3[1.0%]). There was no significant difference in the total daily dose of insulin used between patients with T2DM with and without COVID-19 infection. Compared with T2DM patients without COVID-19 infection, patients with COVID-19 were less likely to use metformin (88[64.7%] vs 10[6.2%]; p<0.01), thiazolidinediones (30[22.1%] vs 0[0%]; p<0.01) and SGLT2 (66[48.5%] vs 5[3.2%]; p<0.01) (**Table 3**).

**Table 3:** Comparison of treatments between COVID-19 infected and uninfected diabetic patients.

		No. (%)		
	Total (n=298)	COVID-19 uninfected (n=136)	COVID-19 infected (n=162)	p value
Total daily dose of insulin, Median (IQR)	–	14.0 (0.0-28.0)	12.0 (0.0-25.3)	0.559
Insulin	192 (64.1)	89 (65.4)	102 (63.0)	0.657
Metformin	98 (32.9)	88 (64.7)	10 (6.2)	0.000218
Glucosidase inhibitors	48 (15.4)	39 (28.7)	7 (4.3)	0.514174
Thiazolidinediones	30 (10.1)	30 (22.1)	0 (0)	0.000027
Sulfonylureas	7 (2.3)	7 (5.1)	0 (1)	0.368240
Glinides	3 (1.0)	1 (0.7)	2 (1.2)	0.092488
DPP4	41 (13.8)	36 (26.5)	5 (3.1)	0.915298
SGLT2	71 (23.8)	66 (48.5)	5 (3.2)	0.000844
GLP1	20 (6.7)	17 (12.5)	3 (1.9)	0.472987

DPP4, Dipeptidyl peptidase-4; SGLT2, Sodium-glucose cotransporter2; GLP1, Glucagon-like peptide-1.

## 4 Discussion

This study was a retrospective study of 298 hospitalized T2DM patients with or without COVID-19 infection, and analyzed baseline data, clinical symptoms, acute complications, laboratory findings, treatment measures and outcome data. From the present results, the severity of T2DM patients with COVID-19 would be higher than those without COVID-19. This may be related to angiotensin-converting enzyme 2 (ACE2), whose expression is elevated in T2DM patients. SARS-CoV-2 binds to ACE2 receptors and can cause multiple organ damage. Moreover, high blood glucose activates inflammatory pathways and increases oxidative damage, impairing immune cell function (13, 14).

In many COVID-19 epidemic studies, COVID-19 with diabetes mostly occurred in the elderly (12, 15)..Recent studies have also indicated that the older the age of T2DM patients with COVID-19 is, the higher the incidence of severe clinical courses and increased mortality (16, 17). In the study, T2DM patients with COVID-19 had a longer duration of diabetes, indicating that this group of people was at higher risk from COVID-19 infection, which was consistent with the characteristics of such patients in other articles (18, 19). The cause was partly attributed to the immune system being impaired due to metabolic inflammation and the body’s ability to deal with infections being reduced in patients with diabetes.

Diabetes is associated with hyperglycemia, and the common symptoms are polyuria, polydipsia, polyphagia and weight loss (20). Consistently, the data of this study showed that these symptoms were the most common in T2DM patients with or without COVID-19. Our study also found that cough was also common in T2DM patients with or without COVID-19, and we speculated that type 2 diabetes may be related to an increased prevalence of respiratory symptoms (21).

Hyperglycemia can trigger an inflammatory response, which leads to structural changes in lung tissue and impaired lung function (22). Such structural changes may also be associated with an increased risk of hospitalization for pneumonia in patients with diabetes (23). The occurrence of fatigue in T2DM patients is also related to the inflammatory response (24), and they showed high levels of inflammatory markers including IL-6, CRP, and neopterin, which plays a role in causing fatigue in T2DM patients (25). Compared to T2DM patients without COVID-19, T2DM patients with COVID-19 had significantly more cough, fatigue, fever, muscle soreness, sore throat and shortness of breath. Studies have shown that patients with COVID-19 have significantly higher numbers of neutrophils (10) and elevated levels of inflammation-related biomarkers (26), which suggests that patients with diabetes are prone to develop an inflammatory storm that ultimately leads to worse symptoms of COVID-19. In addition, oxidative stress caused by persistent hyperglycemia was considered to be the main cause of lung injury in diabetes (27). Patients with diabetes tend to have lower forced vital capacity (FVC) and forced expiratory volume within one second, as well as lower diffusion capacity, which contributes to more severe COVID-19 symptoms (28). Dizziness and nausea were also common in T2DM patients with or without COVID-19, which could be partly explained by diabetic autonomic neuropathy and hypoglycemia caused by T2DM (29, 30). Compared with T2DM patients without COVID-19, hyposmia and hypogeusia were almost exclusively found in COVID-19 patients. Previous studies have shown that 41% and 38% of patients with COVID-19 have hyposmia and hypogeusia, respectively (31). The exact mechanism of hypogeusia and hyposmia in COVID-19 infected patients is not clear, and studies have noted that it may be related to the neuroinvasive potential of SARS-CoV-2 (32). Interestingly, the eating habits of T2DM patients with COVID-19 also changed - they became more polyophagous. This may be related to the COVID-19 lockdown and hyposmia. In a study from Italy, patients experienced a significant increase in appetite due to disruptions in daily work due to the COVID-19 lockdown and stress caused by reading news about COVID-19 from the media (33). Another study showed that patients were unable to perceive taste and flavor, resulting in no sensation of satiation and thus increased appetite (34).

Laboratory findings indicated that the level of creatinine significantly increased in patients with COVID-19, suggesting that kidney damage may have occurred. In a previous cohort study of 5,449 patients admitted to the hospital with COVID-19, 1,993(36.6%) patients developed AKI (35). However, the exact mechanism of COVID-19 on the kidney is unknown, and may be related to the direct damage of SARS-CoV-2 to renal tubules. SARS coronaviruses (including SARS-CoV-2) are detected in urine by PCR, which indicates that the virus interacts directly with or is exposed to renal tubules (36, 37). Furthermore, ACE as a viral receptor is only expressed in the proximal renal tubules, which is parallel to the damaged site of the kidney in patients with SARS-CoV infection (37, 38). Although the median values of creatinine in our study were still within the normal range, they were far from the extent of AKI. However, the difference between the exposure group and the non-exposure group was large, and the present study identified elevated creatinine as a significant predictor of all outcomes of interest (mortality, ICU admission and intubation), which needs to be considered (39).

We found higher ketones and higher rates of diabetic ketoacidosis in T2DM patients with COVID-19 infected, as has been reported in other research. Ketosis occurred in 6.4% of patients with COVID-19 and increased to 11.6% in patients with COVID-19 and diabetes, resulting in a higher mortality rate (33.3%) (40). In a CORONADO study, 11.1% of participants reported diabetes-related disorders at admission, including 40 cases of ketosis, 19 of which were ketoacidosis (2). ACE receptors are expressed in pancreatic tissue and β-cells, and SARS-CoV-2 has been found to bind to ACE2 receptors. Therefore, the metabolic disorders, including DKA may be caused by decreased insulin secretion due to severe insulin resistance and β-cell dysfunction (41, 42). For an unusually high number of patients with COVID-19 developing diabetic ketoacidosis and a hyperosmolar hyperglycemic state, a guideline has been released for the management of DKA (43).

In fact, T2DM patients with COVID-19 are most recommended to be treated with insulin (44). However, our results showed no significant difference between the exposure group and non-exposure group, which may be related to the fact that the proportion of hospitalized patients using insulin therapy was already quite high. From the reported results, T2DM patients with COVID-19 were less likely to use metformin, sodium-glucose transporter-2 inhibitor (SGLT-2i) and thiazolidinediones than patients without COVID-19 infection. This may be associated with insulin therapy reduced expression of ACE2, while metformin, glucagon-like peptide-1 agonists and thiazolidinediones up-regulate ACE2 expression (45). Furthermore, practical recommendations indicated that discontinuing of metformin and SGLT-2i is recommended in patients with diabetes who have a severe course of COVID-19 (42). In randomized controlled trials, the risk of DKA after SGLT2 use was two times higher in patients with T2DM than in controls (46). In our results patients with DKA and increased creatinine were numerous in the exposure group and the use of metformin required close monitoring for acidosis and decreased renal function, so there was a decrease in metformin use. However, previous studies have shown that metformin and SGLT-2i are associated with reduced mortality in patients with COVID-19 and type 2 diabetes, possibly due to reduced release of inflammatory cytokines, so metformin and SGLT-2i can be used for asymptomatic and mild COVID-19 patients (42, 47). Thiazolidinediones have been found to reduce markers of inflammation in COVID-19 patients (48). However, as a second or even third line treatment, metformin is not explicitly recommended for T2DM patients with COVID-19, which may be the reason why it was not used in the exposure group in our study.

The strengths of this study are that it was a multicenter study with an adequate sample size and comprehensive clinical records. Furthermore, to the best of our knowledge, this is the first study to investigate the clinical characteristics and outcomes of hospitalized COVID-19 patients with diabetes in southern China since the COVID-19 policy adjustment. Our study also has certain limitations. First, it is difficult to assess risk factors for poor prognosis due to short-term outcome follow-up. Second, cases with mild symptoms who were treated at home were missed, so this study only represents patients with more severe COVID-19.

## 5 Conclusion

In conclusion, COVID-19 patients with diabetes showed more severe respiratory and constitutional symptoms and an increased proportion of hyposmia and hypogeusia. Moreover, T2DM patients with COVID-19 have a higher incidence of acute complications, are more prone to worsening renal function, and are more cautious about the use of antidiabetic drugs.

## Data availability statement

The raw data supporting the conclusions of this article will be made available by the authors, without undue reservation.

## Ethics statement

The studies involving humans were approved by the Fifth Affiliated of Guangzhou Medical University Research Ethics Committee (GYWY-L2023-64). The studies were conducted in accordance with the local legislation and institutional requirements. The participants provided their written informed consent to participate in this study.

## Author contributions

C-pZ and W-pS performed the general development and design of the study and contributed to critical revisions. X-yZ is the first author who contributed to the design, statistical analysis and manuscript writing of the study. S-fH wrote sections of the manuscript. S-fH, J-xL, H-nZ, LX, X-zW, K-hG, LZ, TL, H-mY, M-rL, and X-yL contributed to data collection and helped perform the analysis with constructive discussion. All authors contributed to the article and approved the submitted version.
